# Mr. Chamberlaine, on the Amendments of the Medicine Act

**Published:** 1803-10-01

**Authors:** W. Chamberlaine

**Affiliations:** Surgeon. Aylesbury Street, Clerkenwell


### Mr. Chamberlaine, *on the* Amendments *of the* Medicine Act.

[ Continued from pp. 258—266. ]

By the 25th clause, the common informer was allowed the scope of six months to bring his information; in which time the mischief he had it in his power to do, was incalculable; as must appear to any one who will only consider how much mischief might have been done, had the informations brought at the Mansion-house in a very short time after the passing of the act, by a common informer, unconnected with the stamp-office, not been quashed; as related in the former part of this account.*

Excepting in the salutary reformation of the Schedule, and the taking the business of information out of the hands of the *common* informer, and confining the power of bringing actions to the Stamp Office, in no case have persons, liable to be affected by this act, greater cause to be thankful to the respectable gentlemen to whom the committee made application for redress, than for the polite attention they paid to their remonstrances, and exposition of the destructive consequences which might possibly ensue, from the allowing the informer, by the 22d clause, six months to make his depredations in, before bringing his informations to issue. The committee represented to Mr. Estcourt, Mr. Vansittart, and Mr. Addington, the possibility of a man being completely ruined by the number of informations laid against him in the course of six months, for the sale of an article which he might erroneously deem not liable to a stamp duty; and they fought hard to obtain a clause in the new act, that the informer should be obliged to sue within ONE month from the time of the penalty being incurred. This ministers did not think themselves warranted to accede to; but, instead of six months, they consented to make it THREE months; and accordingly the 5th clause of the Amendment provides, that every information under the Medicine Act, shall be commenced *within* THREE *months after the offence committed, and not afterwards*.

Considering the very great disproportion of the penalty to the offence, it was represented, that even five pounds, the mitigated penalty, was too much to pay for the omission of a three halfpenny stamp; in reply to this it was argued,

* See page 170 of this volume, (No. 54) for August, 1803.

gued, that if a five pounds penalty attached to this, the omission of a twenty shilling stamp subjected the offender to no greater penalty. It was then suggested by one of the committee to lessen the penalty on the smaller stamps, and lay a fine ad valorem, that is, that the penalty should be in proportion to the value of the stamp omitted to be affixed.

This was objected to as being a matter that would make the act too complicated; and perhaps puzzle the Justices.—However, Government did better for us than we proposed for ourselves, by allowing the magistrate the power of mitigating the penalty to ONE-FOURTH, instead of ONE-HALF, as it before stood. (*Amendment,* latter part of clause 5th.)

It now only remains for me to take notice of that part of the act which relates more nearly to practitioners in Pharmacy, whether concerned in the sale of Quack Medicines or not; in order to shew to both how far they are exonerated from their former fears, and in what cases caution is necessary. And this brings me to the

### SCHEDULE.

It would be the height of absurdity to suppose, that the mechanical parts of acts of parliament are constructed by any of those who compose the great legislative body of the nation.—They are, for the most part, composed by persons who are, or ought to be, conversant in the business which the acts themselves relate to.—But the many incongruities, and the introduction of so many articles which had no business there, into the Schedule of the Medicine Act, by the person who took on himself the trouble of favouring government with that ingenious compilation, shew that

*EX QUOVIS* LIGNO *NON FIT LEGISLATOR.*

The first material alteration we find is, that almost every article in the former Schedule, not clearly defined, has been in the new Schedule omitted; such as *chalybeate pills, carminative tincture, nitre drops, sweating powders, tonic pills,* and many others.

*Arrow root,* or *Indian arrow root.* This, as being more properly an article of *food* than *medicine,* and also an *unmixed* powder, (save when adulterated with potato starch, or flour, by our ingenious sophisticators) is very justly exempted from the stamp duty.

*Arquebusade water.* So great is the repugnance towards paying for stamps, that I once heard a man declare, that if if life could exist without his wearing his head, he would have his own head cut off sooner than be obliged to wear a stamp upon his crown. Another, equally inimical to wearing a stamp upon his head, but more ingenious, bought a second-hand turban that had belonged to one of the Duke of York's black cymbalists, which he wore in going about his grounds on week days, and kept a hat, made before the stamp duty on hats took place, to go to church in, on Sundays.

When the perfumery tax was in existence, government lost *eight thousand pounds* per annum on the importation duties on foreign articles of perfumery. Every one knows how soon the fine powdered head was converted into the black Brutus; and the perfumery tax was ultimately given up, because the cost of collecting it exceeded the sum which the tax itself produced.

I wish a similar loss to government may not be found to ensue, from the laying a stamp duty on *arquebusade water.* This composition already pays a duty of twenty-seven shillings per gallon; it is sold in pint bottles at 7s. 6d. or 8s. each. The stamp duty on each pint is one shilling, which increases the duty on the gallon to eight shillings more, or *one pound fifteen shillings* per gallon duty in all! This enormous duty must rather operate as a prohibition, the good Lady Bountifuls in the country, among whom this article principally finds a sale, will grudge to pay a shilling extra on the pint, for what they consider to be of no use to them or any one else; and will soon find some cheaper substitute to wash their neighbour's wounds with. And when we also take into the scale, that arquebusade water neither is, nor ever was, a secret nostrum, (the composition is given in foreign dispensaries, and also in Lewis's dispensary) we may fairly infer, that this is not a fair article of stamp-*ation,* and that government will lose more than will be gained by bringing it within the meaning of the Medicine Act.

*Blistering ointment, Goulard's extract, Huxham's tincture of bark, Spanish juice, syrup of Tolu, Turkey rhubarb,* being all articles of the London Pharmacopœia, are very properly omitted. *Eau de luce, essence of peppermint,** and *refined liquorice,* are also expunged. *Candied horehound* and *candied ginger,* and a great many articles clearly belonging

* If sold simply in a common phial, but if sold "in quackish guise," it is liable. Juniper's essence of peppermint is particularly mentioned.

longing to the province of the confectioner, but for the sale of which, many grocers, confectioners, and apothecaries have been heavily mulcted under the old acts, are now expunged.

In the former schedule, the words, "OF ALL SORTS," were annexed to the following articles: *Dentifrices, issue plasters, lozenges, lip-salves, tooth-powders, tinctures for the teeth and gums;* thus lumping into one mass every thing that could be construed to answer any of the above purposes; which made it dangerous to an apothecary to sell a little powder or tincture of myrrh, or a small box of sperma cæti ointment, lest it should be purchased by an informer, who should call it lip-salve.

In the new schedule, not only is this exceptionable, sweeping expression, "OF ALL SORTS," done away, but every one of these articles, Lozenges excepted, may be safely sold, provided not sold under the name of any person claiming a proprietary right, or pretended secret, in them, or setting forth a superiority of his own over all other articles of the same kind, by printed hand-bill or advertisement.

In the new schedule, we find an article that I never heard of before, viz. *Chamberlain's ointment*; and I merely notice it here, to say, I am very sorry that any one, bearing that name, should be author of a quack medicine; (I know it is not my friend Chamberlain, the druggist, in Fleet-street;) and I am sorry on another account, because it might be supposed by those who do not know me, that the active part which I am proud to say I have taken in this business, was from interested motives; I therefore take this opportunity of saying, that I know nothing of either the ointment or its author; that I deal not in nostrums, and that I mortally abominate all manner of Quackery.

The names of a great number of articles, not medicinal, and which appertained chiefly, I might say exclusively, to the perfumer and confectioner, have been removed, in consequence of the petitions presented, and the proper measures taken, by deputations from these bodies. But there is one species retained, *partly* medicinal, but rather more an article coming within the province of the confectioner, although largely dealt in by both the druggist and apothecary, upon which something is to be observed: I mean, LOZENGES.

Although that phrase of universal proscription, "OF ALL SORTS," is not, in the new schedule, applied to the article, LOZENGES; yet there are very few of these, which have any any thing medicinal in their composition, that are not specified in the schedule. Such as bear the name of any particular person; as, *Ching's, Dawson's, Steers's, &c.* as also such as are sold as secrets under any fine name; as, *Lozenges of Blois, Patirosa;* or any expressing the quality or the complaint they are good for; as, *paregoric lozenges, worm, heartburn,* or *tooth-ach lozenges,* are clearly within the meaning of the act. These there can be no dispute about; but there are several sort of lozenges prepared, which never were, by any person whatever, pretended to be kept secret, as to their composition; for instance, peppermint lozenges, ginger, horehound, and some others, which are, nevertheless, inserted in the schedule.

At first, it seemed the intention of government to expunge the article, Lozenges, altogether; and if that omission had taken place, there would have been no loss by it, because the 19th clause would effectually secure the duties upon all such as should come under the description of secrets in the hands of particular persons. But, as they are retained, and specifically mentioned by their different names, the dealers in that article are placed in a very unpleasant situation.

It has been already observed, (page 260) that the consumption of lozenges made in England takes off a quantity of the best double refined sugar annually to the amount of not less than one hundred and seventy tons weight; and the preparing the different sorts of lozenges gives employment to a great number of people who are called lozenge makers, and who make nothing else.

The Committee were very strenuous in their endeavours to have an exemption for peppermint lozenges and a few others of very general consumption, and not classed in the rank of nostrums. Possibly they might have succeeded, but the very great press of business of the utmost national importance, which at that time occupied the attention of ministers prevented an opportunity of a discussion on this subject, with them; but the Committee received from the Solicitor of the Stamp Office, an assurance, "That lozenges, sold in ounces, half-ounces, &c. should not be considered as chargable with the stamp duties, provided those lozenges were sold without any printed or written paper or hand bill, expressive of their properties or virtues in the cure of any disease; but if any paper whatever, describing their properties, should be delivered with them; or if any person should vend lozenges, generally understood stood to be, or to have been formerly,* secret compositions in the hands of particular persons, such would certainly be deemed liable to the duty."

This, as I have said before, is a very small boon, and places the dealers in this article, wholesale as well as retail, in a very awkward situation.

If peppermint lozenges are a simple article of confectionery, never claimed as a secret by any one, they should have been exempt from a stamp duty, which will bear very hard on the wholesale dealers and those who sell in larger quantities than an ounce; *little* dealers are allowed, *speciali gratiâ,* and as an indulgence, that which both little and wholesale dealers should have enjoyed *de jure,* an exemption from stamps on that article, and a few others in the same predicament; but in the hurry of national concerns, it is impossible to give attention to minutiæ.

*Issue plasters,* which were in the former schedule, are left out; informations under the old act were always quashed by the magistrates, as it seemed agreed that these were no more than a *bandage,* and not a medicine. But if sold with a bill, intimating that they cure corns, or have any other specific virtue, they are certainly liable. This it is proper apothecaries should be apprized of, as they are an article sold by every apothecary who keeps a retail shop.

*Plaster, black, Court Plaster,* is not in the schedule, and may be safely sold unstamped, although sold with stamps

* Tolu *Lozenges,* for instance. Every one knows how to make these; but the informers have made, under the old acts, a greater harvest out of this one article, than all other medicines put together; and many thousand pounds have been wrung out of the pockets of poor apothecaries who were ignorant that these were a stampable article. What led to the Tolu lozenges being a favourite bait with informers, was, the circumstance that the magistrates themselves were divided in their opinion; for, at one office, magistrates would not convict, because no one pretended it was a secret; whereas, at the next office, the justices remembered that it had once been a patent medicine, and therefore conviction followed of course.

This villainous tribe of gentry, taking advantage of this difference of opinion, when they had marked any one for their prey, used to purchase a box of Tolu lozenges and lay their information at some office where they knew it would be quashed. After thus plausibly inveigling the apothecary into an opinion that he might be perfectly safe in future in selling his Tolu lozenges without stamps, in some short time after, different faces, belonging to the same gang, would come to the same shop, and purchase two, six, or perhaps a dozen boxes, one at a time; and then lay separate informations for each box; making sure it should be at some office where they knew from past experience, conviction would be certain. Many hundred apothecaries have been robbed in this manner.

stamps by some who find an interest in getting off as many as ever they can, as the discount on taking very large quantities renders the stamps a very profitable article of *trade* to them.

*Relfe's suckling assistant.* A mechanical contrivance, and one of the most admirable ever devised for the relief of that part of the creation from whom we derive our greatest comforts, and who merit all our kindness and attention at all times, but especially when labouring under that most painful and distressing complaint, sore nipples. This was one of those articles of the schedule which, though not belonging to the Pharmacopœia, I took the liberty of animadverting upon, and marking as an improper subject of taxation. The inventress, whom I understand to be a sensible and skilful midwife, rather merited a handsome reward for her ingenuity, than to have a tax, and that, one that confers disgrace, imposed on her invention. As well might umbilical trusses, wooden legs, artificial noses, silver palate-pieces, laced stockings, fleecy hosiery, and every item of the stocks in trade of Mess. Sleath, Eddy, Sheldrake, and every other disciple of Taljacotius, be deemed quack *medicines*, and rendered liable to the stamp duty. Mrs. Relfe's machine is very properly exempted, since the act has been amended, from the stamp duty.

*Red pills. Tooth-ach pills.* So great was the panic struck by the act in its unamended state, that I positively know an apothecary who lost a few shillings by refusing to make up a box of pills, in which cinnabar. factit. was a principal ingredient, for fear the customer should inform against him for selling *red pills*; and the same person feared to dispense an opium pill to a stranger, lest it might cost him ten pounds on its being sworn to being sold as a TOOTH-ACH PILL! These, and as I have before observed, most other medicines indefinitely described, are omitted, and no danger attaches, unless the vender characterizes such as empirical remedies.

*Troches of all sorts.* To the apothecary who is attached to this almost obsolete form of medicine, it may be interesting to know that the omission of the word *troches* altogether, in the amended schedule, wholly exonerates him from any fears on that score.

*Tinctures for the teeth and gums.* By some oversight, in the hurry of other business, the above words stand part of the new schedule; but as the committee have received assurances that the commissioners of the stamps will never encourage any vexatious suit, nor suffer the act of parliament to be strained beyond its just and fair limits, no apprehensions need be entertained by the apothecary acting in the fair and regular way of business.

The assurances already stated to have been given by the Solicitor of the Stamp-office on the part of the Commissioners, renders it unnecessary for me to specify every item of the old schedule, exonerated by that which is now in force, from a stamp duty. It may however be expedient for those whom it concerns, to possess *both* schedules; accurately mark wherein they differ, and particularize in the latter, such articles as they may be dealers in.

In taking a summary view of the whole, I have to congratulate the regular practitioner in pharmacy, and the retail apothecary in particular, upon his liberation from the dangers and apprehensions which the act of 1802 subjected him to; and all this, *free of expence*, as I may say: the druggists, who are not quite so far benefited, having borne nineteen twentieths of the expence of getting the business of the AMENDMENT through parliament; comprehending,

1. The exemption of *unmixed* drugs from a stamp duty.
2. The taking the business of prosecution out of the hands of *common* informers, and confining it to the Stamp-office and Attorney-general.
3. The obliging the informer to bring his action in *three* months instead of *six* months.
4. The allowing the magistrate to mitigate the penalty to *one fourth* instead of *one half*; and,
5. The exclusion of all articles from the schedule, indefinitely expressed, and which would, by being retained, endanger the practice of the regular medical practitioner.

The druggists, however, still labour under some hardships and inconveniences: In compliance with the wishes and instructions of their constituents, the committee essayed for a total repeal.—Had the druggists been less intent on obtaining this, and testified a greater readiness to give assistance to ministers in framing an amelioration of the act, the few exceptionable parts of the act, which still remain, would have been done away.

It would be injustice to conclude this paper, without paying a tribute of thanks to those Gentlemen, to whom in the course of the business, it was necessary to make application.

The Commissioners of the Stamp-office were easily accessible, and their deportment towards those who applied to them was marked by that liberality which characterizes the the gentleman. To Mr. Addington and Mr. Vansittart, many thanks are due for the readiness with which they granted conferences, and the attention they paid to what was laid before them by the deputations of the committee appointed to wait on them; as also to Mr. Estcourt, Solicitor of the Stamp-office, for his very great attention and patient consideration of every particular in the many interviews held with the members of the committee.

To the Lord Mayor, (Mr. Alderman Price) to Mr. Alderman Combe, and to Sir John William Anderson, members for the City of London, and to Isaac Hawkins Browne, Esq. M. P. for their easiness of access at all times, their polite attention, their patient hearing, their useful advice, and their friendly support in parliament, that gratitude, which on all occasions they have a just claim to from their fellow-citizens, is, on this occasion, most justly their due!

I am, &c.

W. CHAMBERLAINE, Surgeon.

*Aylesbury Street, Clerkenwell,*
*August* 12, 1803.

